# Stressful life events and prenatal representations of the child

**DOI:** 10.1080/14616734.2024.2345242

**Published:** 2024-04-24

**Authors:** Lauren G. Bailes, Abigail Blum, Whitney Barnett, Hannah Piersiak, Sydney Takemoto, Brooke Fleming, Caelan Alexander, Kathryn L. Humphreys

**Affiliations:** Psychology and Human Development, Vanderbilt University, Nashville, USA

**Keywords:** Mental representations, prenatal representations, stressful life experiences, pregnancy stress, childhood stress

## Abstract

Caregivers’ mental representations of their children can be assessed prenatally and are prospectively associated with later caregiving quality and caregiver–child attachment. Compared to balanced, distorted or disengaged representations are linked to insecure caregiver–child attachments. The present study explored factors (i. e. stressful life experiences and positive experiences) that may be linked to risk for distorted and disengaged representations. We used a brief version of the Prenatal Working Model of the Child Interview in a sample of 298 pregnant people (ages 19 to 45 years; *M* = 30.83, SD = 5.00) between gestational age 11–38 weeks (*M* = 23.49, SD = 5.70). A greater number of stressful events across three developmental periods (i.e., lifespan, childhood, and pregnancy) were related to increased odds of distorted, compared to balanced classification. Pregnancy stress had the largest association. Positive experiences from childhood did not buffer the association between stress and representations. Findings highlight the importance of stress on prenatal representations of one’s child.

Theoretical and empirical work has suggested that caregivers’ thoughts and feelings about their children influence caregiving behavior (George & Soloman, 1999; Leerkes & Augustine, 2019; C. Zeanah et al., 1994). Caregivers develop internal working models, or mental representations, of their children (C. H. Zeanah & Anders, 1987). One of the most frequently used assessments of caregiver internal working models, both pre- and postnatally, is the Working Model of the Child Interview (C. Zeanah et al., 1994), a semi-structured interview that asks caregivers to describe their child and their relationship with them. According to Zeanah & Benoit (1995), representations from the WMCI can be classified into three categories: balanced, disengaged, or distorted. Balanced representations are characterized by coherence, flexibility (or openness to change), and richness. These signal that the caregiver values their relationship with their child and acknowledges the importance of that relationship (Benoit et al., 1997). Disengaged representations are characterized by emotional aloofness or pervasive distancing from the fetus or infant and tend to reflect a lack of personal investment with the child (Benoit & Parker, 1997). Distorted representations, though characterized by a higher degree of involvement than found in disengaged representations, tend to reflect that the caregiver does not convey a clear sense of the infant. This lack of clarity may be due to several types of distortions, including self-involvement, distraction, role-reversal, or confusion in relation to the infant.

Relative to the other representations, balanced representations are linked to higher infant attachment security scores (Ahlfs-Dunn et al., 2022; Benoit et al., 1997; Hall et al., 2015; C. Zeanah et al., 1994) and greater child positive affect during caregiver – child interactions (Rosenblum et al., 2002). Disengaged and distorted representations have been linked to child difficulties, including more negative mood and lower attention and play skills during interactions (Korja et al., 2010; Rosenblum et al., 2002). Non-balanced representations are also associated with difficulties in child affect regulation and behavior modulation (Benoit et al., 1997; Dollberg et al., 2010) and toddler internalizing and externalizing behaviors (Gravener et al., 2012; Sher-Censor et al., 2018).

Internal working models of the child begin to develop prenatally. Although most studies have examined caregiver representations of their children postnatally, representations formed prenatally are associated with both child and dyadic functioning (Ahlfs-Dunn et al., 2022; Guyon-Harris et al., 2022). For instance, Guyon-Harris et al. (2022) found that children with mothers classified in pregnancy as having a non-balanced prenatal representation had, on average, a greater number of socio-emotional difficulties at age 24 months, suggesting a potential long term association of prenatal representations on child development. These prenatal representations of the child (hereafter, prenatal representations) have been posited to relate to the affective organization of mood, social context, and social support during pregnancy (Ahlqvist-Björkroth et al., 2016; Stern, 1995). Specifically, Stern (1995) proposed that pregnant people tend to organize and process their own affective experiences about pregnancy by drawing on their moods, social context, and supports that they receive during pregnancy.

Considering that prenatal representations are a risk factor for insensitive caregiving (Guyon-Harris et al., 2022) – a role that may be causal, and importantly, modifiable – it is essential to examine factors that may influence prenatal representations. Once such factor that has been examined extensively with respect to postnatal representations is caregiver stress (e.g. Huth-Bocks et al., 2004; Malone et al., 2010). However, it is possible that caregivers’ experiences with stress may be related to their emerging prenatal representations of their unborn child. If so, the window of opportunity for intervention and prevention focused on the caregiver – child relationship can reasonably include the prenatal period when, perhaps, caregivers may be more open to receiving intervention efforts (Jeong et al., 2021; Kitzman et al., 1997).

Although we expect stress may have important implications for caregivers’ emerging representations of their children, there may also be factors that buffer the negative effects of stress on parenting cognitions. Specifically, “angels in the nursery,” or positive memories and recollections from childhood (Lieberman et al., 2005), may support the development of adaptive and balanced representations and attachment. Childhood experiences of acceptance, love, and a sense of security and self-worth may provide a buffer between adverse life experiences and the relationship with one’s child (Han et al., 2023; Lieberman et al., 2005). The resilience literature has centered on the protective role of positive childhood experiences as a buffer for the negative associations of stress and early life adversity on children’s adjustment (Narayan et al., 2018). Further, finding that this buffering effect may last into adulthood (Narayan et al., 2018). Specifically, these studies have found that among those with a history of adverse or stressful life events, more positive experiences are associated with stronger relationships in adulthood (Powell et al., 2021) and reduced risk for depression (Bethell et al., 2019) and posttraumatic stress (Narayan et al., 2017). These positive experiences provide resources in adulthood that can be drawn upon to foster affection, communication, trust, and strong social support networks, which may potentially buffer the impact of stressful life events.

## Present study

The present study had three main aims: (1) examine the association between stressful life events and prenatal representation classification; (2) examine two potential sensitive periods (i.e. during childhood and during pregnancy) in which stress may be associated with prenatal representation classifications; and (3) examine whether positive childhood experiences moderate the association between stressful events during childhood and prenatal representation classifications. We hypothesized that, overall, any stressful life events would be associated with a higher likelihood of being classified as disengaged and distorted, compared to balanced. We also hypothesized that stressful events during pregnancy, in particular, may be associated with prenatal representations, such that participants who endorse more stressful experiences in pregnancy will be less likely to have balanced prenatal representations compared to those who endorsed fewer stressful experiences (Pajulo et al., 2001). We expected that stressful events during pregnancy may be particularly related to less balanced representations given this is a period in which the parent – child relationship first develops (Ramsdell & Brock, 2021). Lastly, we expected that stressful life events during childhood would be associated with a higher likelihood of being classified as non-balanced (i.e. distorted or disengaged) compared to balanced. However, we hypothesized that positive childhood experiences would reflect the potential buffering of this association, such that the relation would be weaker with more positive childhood experiences.

## Method

### Participants

Participants were drawn from a longitudinal study from a large metropolitan city in the central southeastern part of the United States. In total, 326 pregnant people were enrolled in the study and provided data during pregnancy. Due to technical difficulties (e.g. interview recording terminated prematurely), 28 interviews were unable to be coded, resulting in 298 participants included in these analyses. These 298 participants ranged in age from 19 to 45 years (*M* = 30.83, *SD* = 5.00). Participants self-reported their race; most participants identified as White (73%), 8% as Black/African American, 4% as multiracial/other, 2% as Asian, and < 1% as Native Hawaiian or Pacific Islander. Seven percent identified their ethnicity as Hispanic. Most participants were married (81% married, 15% single, and 2% divorced/separated) and employed for wages (78% employed for wages, 4% homemaker, 6% student, 3% out of work looking for work, 2% self-employed, 7% other). Income to needs ratio ranged from 0 to 3 (*M* = 1.70, *SD* = 0.76; 24% had ratio < 1). Fifty-six percent of our sample were first time parents, 33% reported having one other child, and 11% reported having two or more children. Participants were recruited from local obstetric clinics, printed advertisements, and social media advertising. Eligibility requirements for participants included (a) US citizenship or permanent residence, (b) 18 years of age, (c) fluency in English, and (d) between 11 and 38 weeks gestation. All recruitment and study procedures performed were in accordance with the prevailing ethical standards. The Vanderbilt University Institutional Review Board approved the study, and all participants provided informed consent prior to participating.

### Procedure

Participants attended an in-lab or remote session (due to the COVID-19 Pandemic) during the second or third trimester of pregnancy (range from 11 to 38 weeks; *M* weeks gestation = 23.49, *SD* = 5.70). At this session, participants completed questionnaires and interviews with clinically trained study team members (interviews and measures are detailed below).

### Measures

#### Working of the model of the child interview

The Working Model of the Child Interview (WMCI; C. Zeanah et al., 1994) is a semi-structured interview designed to assess caregivers’ representations of their relationship with a specific child. The WMCI has been repeatedly validated as a useful clinical and research tool for examining the role of caregiver representations in child development (Benoit et al., 1997; Vreeswijk et al., 2012), including prenatally via the Prenatal WMCI. The present study implemented a shortened version of the interview (the WMCI-Brief), which consisted of 10 questions (items are available on OSF Link; https://osf.io/duakq/). In consultation with Dr. Zeanah, this brief interview was intended to take approximately 20 minutes to administer and capture the relevant constructs to enable coding via the Working Model of the Child Interview Coding Manual (Zeanah et al., 1996). All 16 continuous scales (e.g. richness of perceptions, coherence, and overall balanced rating) as well as classifications into one of the three representation types (i.e. balanced, disengaged, or distorted) and subtypes were coded (for classifications within the study see Table 1). Each interview was coded by at least two coders to minimize potential bias and increase reliability. If coders scores were more than 1 point apart on at least 5 of the 16 subscales (14% of interviews) or if coders disagreed on classifications, a third coder was assigned to review the interview. Final codes were derived via consensus among all coders (i.e. either two or three coders) assigned to the interview. Post-consensus agreement between consensus scores and a single randomly selected rater was high (80%; weighted Kappa = .64), indicating that individual coders were, on average, providing similar determinations as was made via consensus. Participants were classified into one of the three types of overarching representations: balanced (60%), disengaged (15%), or distorted (25%; Zeanah et al., 1996). This is relatively consistent with Vreeswijk et al. (2012) review rates in non-clinical samples of 53% balanced, 21% disengaged, and 26% distorted.

#### Life stress interview–revised

The Life Stressor Checklist – Revised (LSC-R) is a self-report interview measure designed to assess lifetime traumatic or stressful events; specifically, this interview includes 29 possible stressful life events (Wolfe et al., 1996). Events include exposure to disasters or accidents (e.g. a tornado, hurricane, car wreck), physical and emotional maltreatment, sexual abuse and harassment, medical issues, deaths, and childhood-specific related stressors (e.g. parental divorce, foster care, physical neglect). For endorsed events (yes/no format), participants were also asked at what age(s) the event(s) occurred. A score ranging from 0–29 was then computed based on the total number of unique lifetime events a participant endorsed. Three variables were created: *lifespan stress* count reflecting the total number of unique stressors that a participant endorsed over their entire life; *childhood stress* count reflecting unique events that occurred during the first 18 years of a participant’s life, and *pregnancy stress* count reflecting unique events that only occurred during this pregnancy (not necessarily stress about the pregnancy). For all three variables, higher scores reflect a greater number of stressful life events. The LSC-R has demonstrated good criterion validity in relation to assessing stressful events in diverse samples (Gavrilovic et al., 2002) and has demonstrated acceptable test-retest reliability (weighted Kappas = .59; McHugo et al., 2005).

#### Benevolent childhood experiences

Participants completed the Benevolent Childhood Experiences (BCE) questionnaire to measure positive and supportive early life experiences (Narayan et al., 2018). This questionnaire is a 10-item instrument that assesses different dimensions of positive experiences from the first 16 years of life, including predictable qualities of life (e.g. *did you have opportunities to have a good time?*), security and support (e.g. *did you have at least one teacher who cared about you?*), internal perceived safety (e.g. *did you have beliefs that gave you comfort*), and external perceived safety (e.g. *did you have at least one caregiver with whom you felt safe?*). Items are binary (0 = no, 1 = yes), and the number of items endorsed are summed together ranging from 0 to 10, with higher scores indicating more positive early life experiences. The BCE measure has demonstrated strong predictive and concurrent validity and high test-retest reliability (test-retest correlations range from .73 to .80; Narayan et al., 2018).

### Data analyses

#### Data availability statement

Data and code used in the subsequent analyses are publicly available (https://osf.io/vc5mr/).

#### Analytic plan

Analyses were conducted in SPSS version 28 (IBM Corp., 2021). Data visualization was conducted in R version 4.1.3 (R Core Team, 2022). Pregnant people’s representations of their unborn child were coded in a variable for balanced, disengaged, or distorted, thus, all aims were addressed using logistic regressions where the number of stressful life events was specified as a predictor of classification (total stressful life events for Aim 1; pregnancy and childhood stressful events for Aim 2). For Aim 3, we included benevolent childhood experiences as a moderator of the association between stressful events from childhood and pregnant people’s representations. For all models, balanced was specified as the reference group, such that for each model, two comparisons were made: balanced versus disengaged and balanced versus distorted. We examined potential covariates including participant age, gestational age, and parity; these covariates were not associated with prenatal representation classification. Although family income may be a theoretically relevant covariate to include, given that some LSCR items assess stressful life events related to financial strain, inclusion of both would remove shared variance explained by the LSCR scores. All continuous variables were mean-centered before being entered into the regression analysis. A product term of the centered BCE and centered childhood stress variable was created for testing Aim 3.

## Results

### Preliminary analyses

**Figure 1.**
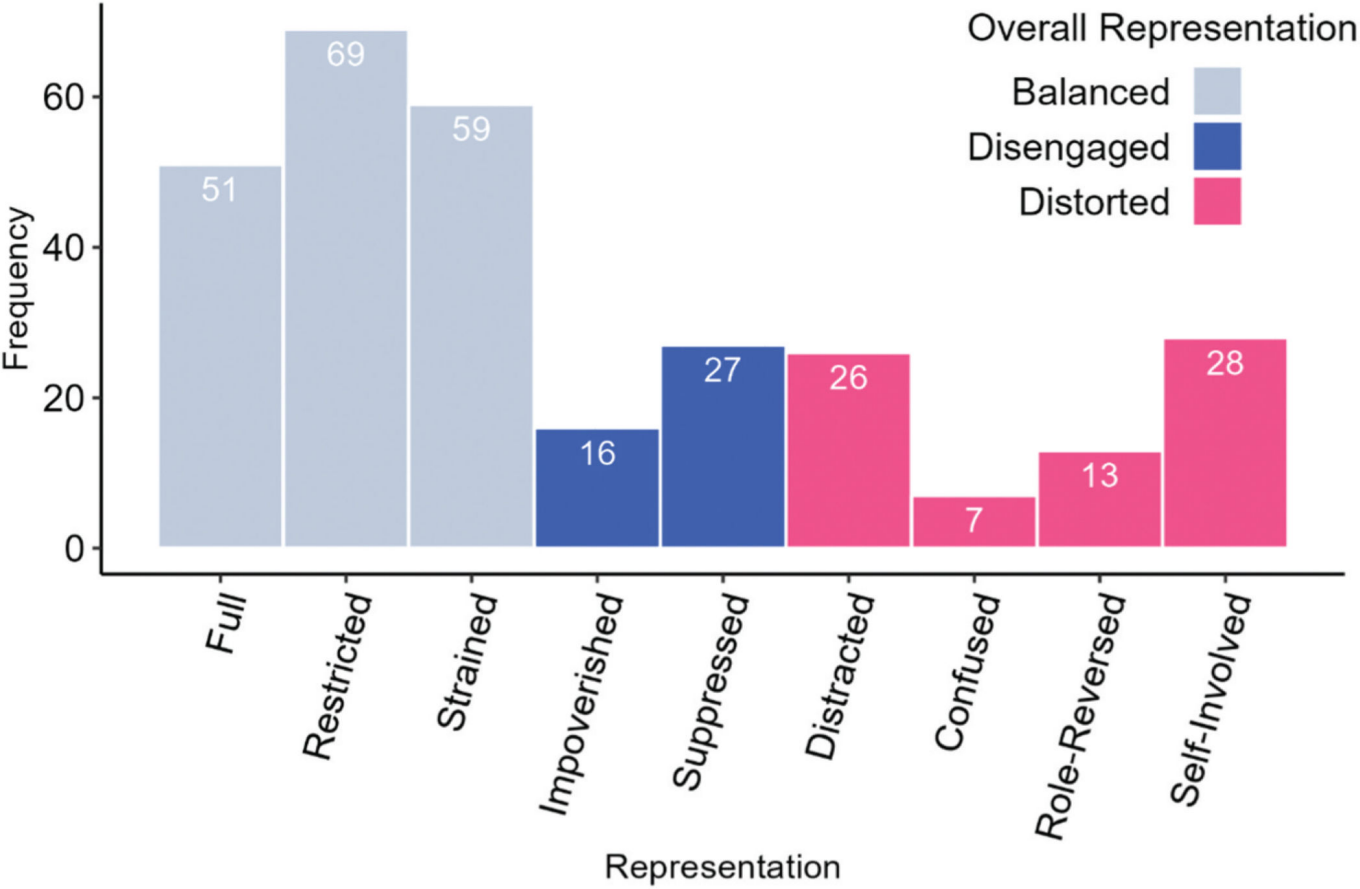
WMCI representation classifications by overall representation and subclassifications. *Note*. Balanced *n* = 179, Disengaged *n* = 43, Distorted *n* = 74.

Descriptive statistics for continuous variables of interest are presented in Table 2, and frequencies of classifications of WMCI prenatal representations are presented in Figure 1. Bivariate correlations among variables of interest are presented in Table 3. Of note, positive childhood experiences were negatively correlated with lifespan stress and childhood stress, but not pregnancy stress. We examined potential covariates including participant age, gestational age, income to needs, and parity. We examined associations between demographic variables and variables of interest. The omnibus ANOVA examining differences in maternal age across classifications revealed that there were differences in participant age, *F*(2, 262) = 6.45, *p* = .002, such that participants with prenatal representations classified as balanced were older, on average, than those with disengaged classifications, mean difference = 3.11 years, 95% CI [1.07, 5.15], *p* = .001. Results did not differ when maternal age was included as a covariate. For model parsimony reasons, we present results without maternal age included. The omnibus ANOVA examining differences in income across classifications was statistically significant, *F*(2, 281) = 8.48, *p* < .001, such that participants who had prenatal representations classified as balanced, compared to disengaged, were higher in income-to-needs ratio, mean difference = 0.52, 95% CI [0.21, 0.83], *p* < .001. Given that some LSCR items capture events related to financial strain, inclusion of income-to-needs ratio would result in shared variance relevant to life stress being removed from the model of the LSCR.

### Primary analyses

#### Aim 1. Examine the association between stressful life events and pregnant people’s representations of their unborn child

**Figure 2.**
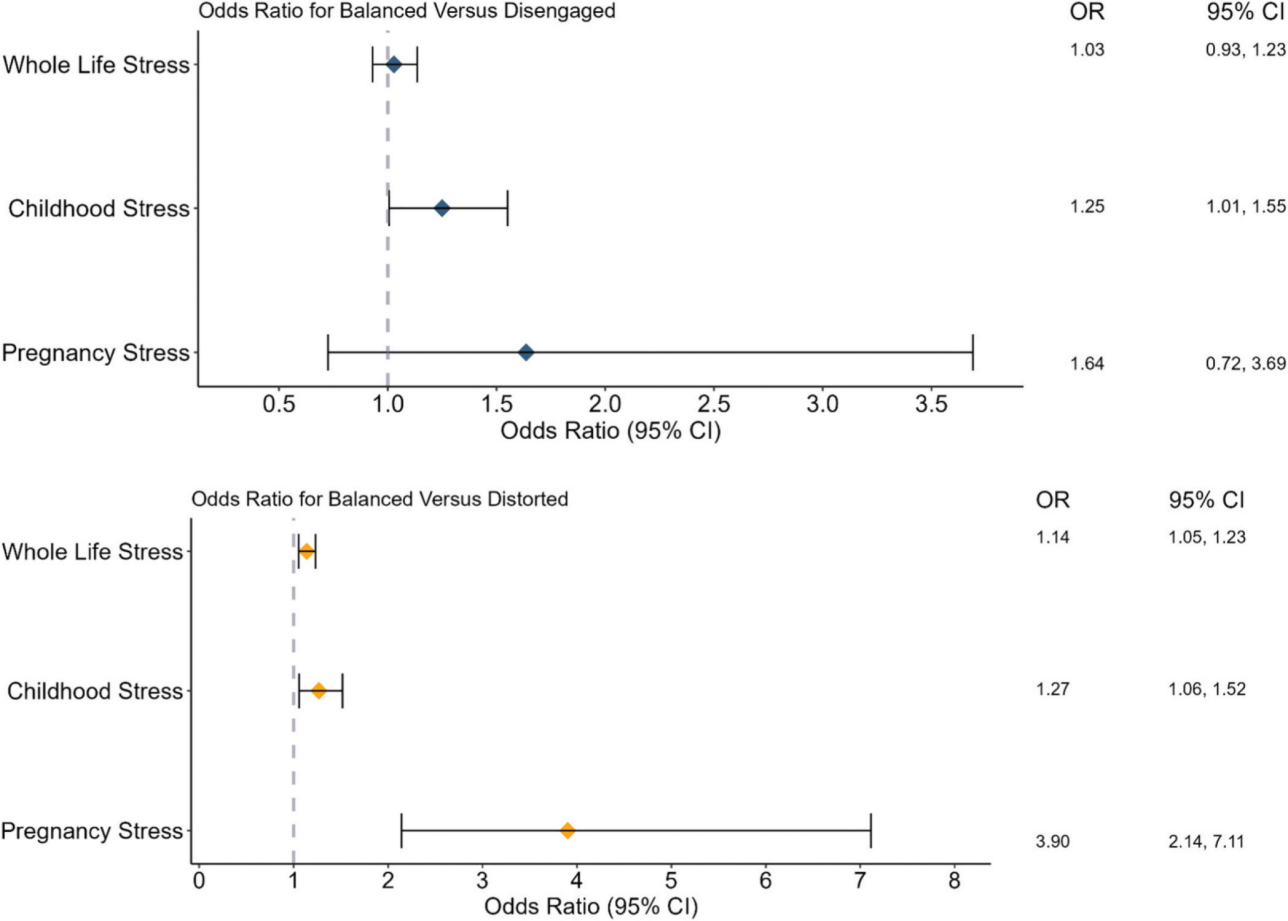
Odds ratio for being classified as balanced vs. disengaged (top panel) and balanced vs. Distorted (bottom panel) by stress.

Aim 1 was tested using a multinomial logistic regression where number of stressful life events were specified as the independent variables and pregnant people’s representations as the dependent variable. The omnibus model was statistically significant, χ^2^(2) = 11.03, *p* = .004. When examining differences between balanced and disengaged classifications (see Figure 2), lifespan stress was not associated with likelihood of classification of balanced versus disengaged. However, lifespan stress was associated with a higher likelihood of being classified as distorted compared to balanced (B = 0.13, SE = 0.04, *p* < .001). Specifically, for each additional life stressor, there was a 1.14 increase (14% increase) in the likelihood of being classified as distorted compared to balanced (OR 95% CI [1.05, 1.23]).

#### Aim 2. Examine two potential sensitive periods for the effect of stress on prenatal representations of the child

The omnibus model examining pregnancy stress specifically on pregnant people’s representations was statistically significant, χ^2^(2) = 24.21, *p* < .001. Similar to lifespan stress model (see Figure 2), stressful events during pregnancy were associated with higher odds of being classified as distorted compared to balanced (B = 1.36, SE = 0.31, *p* < .001), such that for each additional stressor, the odds of being classified as distorted increased by 3.90, or 390% (95% CI [2.14, 7.11]).

The omnibus model examining childhood stress specifically on pregnant people’s representations was statistically significant, χ^2^(2) = 8.34, *p* = .016. Stressful events during childhood were associated with higher odds of being classified as distorted compared to balanced (B = 0.24, SE = 0.09, *p* = .010), such that for each additional stressor, the odds of being classified as distorted increased by 1.27 (95% CI [1.06, 1.52]).

To examine the unique associations of pregnancy stress or childhood stress on pregnant people’s representations, we entered both as independent variables into a logistic regression model (see Figure 2). The omnibus model was statistically significant, χ^2^(4) = 28.68, *p* < .001. Neither pregnancy stress nor childhood stress was related to higher odds of being classified as disengaged compared to balanced. However, in a model with both variables included, pregnancy stress was uniquely associated with higher odds of being classified as distorted compared to balanced after accounting for the other (pregnancy stress: B = 1.28, SE = 0.31, *p* < .001). For each additional stressor during pregnancy, the odds of being classified as distorted increased by 3.58 (95% CI [1.94, 6.62]). Childhood stress was not statistically significantly associated with a higher odds of being classified as distorted compared to balanced (B = 0.15, SE = 0.10, *p* = .119).

Follow-up exploratory analyses examined the four subtypes of distorted classifications: distracted, confused, role reversed, and self-involved. Importantly, the cell size for each subclassification differed, and was below recommended cell size cutoffs (Beunckens et al., 2008). These analyses are exploratory and should be interpreted as such. Logistic regression models suggested that pregnancy stress was associated with higher odds of being classified as distorted – distracted compared to all other subtypes combined (B = 1.24, *SE* = 0.33, *p* < .001), such that for each additional stressor reported during pregnancy, the odds of being classified as distorted – distracted increased by 3.47 (95% CI [1.81, 6.63]). Childhood stress was associated with higher odds of being classified as distorted – self-involved compared to all other subtypes combined (B = 0.29, *SE* = 0.12, *p* = .016). For each additional childhood stressor, the odds of being classified as distorted – self-involved increased by 1.34 (95% CI [1.06, 1.69]). Stress, at either time period, was not associated with distorted – confused or distorted – role reversed. Further, using generalized estimating equations and negative binomial distributions, we estimated the difference between stressful life events during childhood and during pregnancy as a function of distorted representation category. Notably, there were no statistically significant differences between number of stressful events during pregnancy (omnibus model not statistically significant; χ^2^(3) = 2.15, *p* = .543) or during childhood (omnibus model not statistically significant; χ^2^(3) = 5.35, *p* = .148) across distorted representation categories subcategories.

#### Aim 3. Examine whether positive childhood experiences moderate the association between stressful events during childhood and pregnant people’s representations

To test the potential moderating effects of BCE on childhood stress and representation, we ran three step-wise models. First, the model containing only covariates and BCE was not statistically significant, χ^2^(2) = 1.50, *p* = .473, suggesting that BCEs did not explain a significant amount of variance in representation classifications. The second model added the effects of childhood stress, and this model was also not statistically significant, χ^2^(4) = 7.37, *p* = .118. Lastly, we included the BCE × childhood stress interaction term to test the potential moderating effects of BCE. This model was also not statistically significant, χ^2^(6) = 8.17, *p* = .226.

## Discussion

In a sample of 298 pregnant individuals, the present study employed a multi-method design, including interviews, self-report measures, and qualitative statement coding, to investigate the relation between pregnant people’s stressful life events and the development of their emerging working model representations of their unborn child. There were four main findings from this study. First, for each additional stressful life event reported by caregivers, there was a 14% increase in being classified as having a distorted representation compared to a balanced representation. Second, stressful events that occurred during pregnancy as well as during childhood were associated with distorted representations, with a particularly pronounced association with stressful events during pregnancy, such that the odds of being classified as distorted (compared to balanced) increased by 390% for each additional stressor during pregnancy. Third, the association between stress and distorted subclassifications were similar, specifically for distorted – distracted and distorted – self-involved. Fourth, we did not find any evidence that positive childhood experiences might mitigate the associations of stressful events during childhood on representations of the unborn child. Findings from the current study provide evidence that pregnancy may be a sensitive time in which stressors may be particularly salient for the development of emerging representations, compared to childhood stressors, although childhood stressors do seem to be playing an important role.

Each additional life stressor that participants reported was associated with increased odds of the pregnant person’s representation being classified as distorted. Previous research has shown that stressful events can be associated with the quality of the emerging caregiver – child relationship, in particular feelings of emotional connectedness toward the unborn child (e.g. Yarcheski et al., 2009; Zhang et al., 2022). Both stress related to (Niccols et al., 2015) and unrelated to (Huth-Bocks et al., 2004) caregiving is linked to distorted postnatal representations. Our study found that the associations between stress and representations are observable even before the child is born, identifying the prenatal period as a time in which the caregiver – child relationship should be targeted for improvement. By focusing on prenatal representations rather than postnatal representations, the current study minimizes potential child driven effects that could be impacting the role of stress on representations.

We considered whether stressful experiences during two periods theorized as particularly salient for representation formation – the participant’s own childhood and pregnancy – are associated with representations about one’s child. Although both types of stress were related to a higher likelihood of being classified as distorted compared to balanced, the effect size was larger for pregnancy stress, suggesting that stress during pregnancy may be especially important for the emerging prenatal representation. Although stressors tend to be correlated throughout the lifespan (e.g. Brennan et al., 2024), and individuals who experience more stress earlier in life tend to develop stress sensitization, such that they may be more responsive to stress later in life (McLaughlin et al., 2010), the present study findings suggest some specificity about risk for stressful events during pregnancy. Thus, finding ways to reduce stressful events during pregnancy and/or buffer pregnant people from the impact of those events represent important targets with the potential for downstream effects on representations of the child. To the degree to which stressful experiences are modifiable, prioritizing such efforts may have positive cascading consequences not only for the pregnant person but also for the developing caregiver – child relationship. Typically, prenatal care centers around promoting the health of the developing fetus and the physical health of the pregnant person. Our findings support other work that emphasizes the importance of reducing stressful experiences during pregnancy to promote positive emerging caregiver – child relationships (Fransson et al., 2020; Manzari et al., 2019).

In addition to the broader distorted categorization, we explored the association between stressful events and representations that are distorted subtypes (i.e. distracted, confused, self-involved, and role-reversed). Both pregnancy and childhood stress were associated with higher odds of representations being classified as distracted or self-involved. We did not find any relation between stress and distorted – confused or distorted – role-reversed, although classifications of these two subtypes were low (confused *n* = 7, role-reversed *n* = 13), which affects power to detect potential effects. Examining the subscales of classifications provides important insight into the potential nuances of distorted representations that may be important for intervention. Characteristics of the subtypes differ, ranging from picturing the child as a best friend and confidant to descriptions that suggest the caregiver is unsure about what their role will be in the child’s life and in the caregiver – child relationship. There is limited empirical work that has examined the specific subtypes of representations in relation to children’s development or the caregiver – child relationship, but there may be important clinical implications in understanding which subtype of distorted representation caregivers may be classified as.

Inconsistent with our expectations, we did not find evidence that stressful events were related to balanced versus disengaged classifications. Vreeswijk et al. (2015) found that prenatal risk was associated with an increased risk for both distorted *and* disengaged, compared to balanced classifications. Importantly, the assessment of prenatal risk in Vreeswijk’s study was much broader than that of the current study, which could, in part, be explaining differences in findings. For example, Vreeswijk et al. (2015) examined factors related to the current pregnancy (e.g. doubts of competence as a parent, whether the child was wanted), social and relational support (e.g. whether the mother was partnered), and external factors spanning timeframes outside of the current pregnancy (e.g. violence and neglect as a child). It is also possible that a cumulative assessment of stressful events may not capture the ways in which some experiences result in a suppression of psychological involvement and/or investment with the child. Stressful events may not suppress psychological involvement and/or investment with the developing infant. Other facets of the caregivers’ environment may be associated with their psychological involvement with their children. For example, caregivers who are at a higher risk for pregnancy loss (e.g. previous history of miscarriage) tend to present a more disengaged prenatal representation (Markin, 2018). Additionally, it is possible that rather than external stressful events, caregivers’ emotional problems (e.g. emotional instability) and psychopathology (e.g. depressive symptoms) may be more strongly associated with disengaged representations (Vreeswijk et al., 2015). Disengaged representations tend to decrease from the prenatal to postnatal period, but prenatal risk factors, including stress, may alter the trajectory from pre- to postnatal representations. For example, Vreeswijk et al. (2015) found that individuals interviewed during pregnancy (i.e. about 26-weeks gestation) that had higher prenatal risk (e.g. moved homes more than twice in the last year, experienced domestic violence) were more likely to remain disengaged when assessed again at infant age 6-months. These findings highlight the importance of examining the change in representations from the pre- to postnatal periods and the potential role of stress in the development of representations over time.

Positive childhood experiences did not appear to buffer the potential negative associations between stressful life experiences and prenatal representations. Although previous research has suggested that positive childhood experiences may be protective against other factors that may undermine well-being (e.g. Lieberman et al., 2005; Narayan et al., 2017), we did not find evidence of this association in the context of representations. One possible explanation could be the distribution of our measure of positive experiences was very positively skewed, which is consistent with other published work (Narayan et al., 2023). The revised BCE measure (Narayan et al., 2023) includes additional items that are less frequently endorsed and may allow for additional variation in scores (e.g. *Did you regularly spend time outside in the sunshine or around nature? Did you feel accepted for who you were?*).

Despite the study’s strengths, the present study has several limitations. We used a binary scoring of stressful life events participants may have experienced throughout their lifetime as opposed to dimensional assessments capturing severity, type, or chronicity. In comparison to subjective measures of stress (i.e. perceived stress) that account for an individual’s subjective experience, counts of experiences allow for the assessment of stressors with less influence on subjective reactions to stress. Although participants in the present study tended to identify as White and were on average highly educated with high income to needs ratios, our rates of classifications were very comparable to non-clinical mothers from a large review conducted by Vreeswijk et al. (2012). Previous work has suggested that these factors are related to subscales of the WMCI. For example, Huth-Bocks et al. (2004) found that demographic risk (i.e. low education attainment, single parenthood) was negatively correlated with richness of WMCI subscales such as richness of perceptions, openness to change, coherence, sensitivity, and acceptance. Thus, it is possible that the pattern of associations related to stress and mental representations may differ among samples with greater diversity in terms of sociodemographic characteristics. Additionally, there is emerging evidence related to the role that positive adult experiences may have in understanding the associations between pregnant people’s experience with stress and mental health and well-being (e.g. Herbell & Zauszniewski, 2019). Although this has yet to be examined in relation to caregiving and the emerging caregiver – child relationship, it is possible that positive life experiences during pregnancy (e.g. high social support, relationship satisfaction) may also buffer the associations between stress and prenatal representations of the child (Moe et al., 2018). This is an important avenue for future work aiming to better understand precursors of the emerging caregiver – child relationship.

Additionally, we used a modified version of the WMCI following consultation with and permission by Dr. Charles Zeanah. The modifications included cutting interview questions in order to ease participant time required to complete the interview. Codes from this shortened version may differ from those obtained via the full interview. Although we tested the relation of stress on subtypes for distorted classifications, the sample size for these tests were small (see Figure 1). Importantly, we only assessed the role of positive experiences from childhood, rather than those from other developmental phases. Although there are theoretical explanations for why positive experiences from childhood may be particularly salient for buffering against the negative effects of stressful life events, there are not well-validated assessments of positive life events throughout the lifespan. However, the possible importance of positive experiences in later life merits additional study given that more time proximal experiences may have greater salience on functioning (Crandall et al., 2023). Lastly, while we did include the three categories of WMCI representations that were originally established by Zeanah & Benoit (1995), more recent research has suggested that there may be a fourth representation that is associated with disorganized attachment, WMCI – disrupted (Crawford & Benoit, 2009). This representation is characterized by dimensions such as role and boundary confusion, fearfulness, disassociation or disorientation when discussing the child, and intrusiveness/negativity toward the child. Many of the characteristics for the disrupted classification overlap with the original distorted representations, suggesting that some participants who were classified as distorted in the current study may meet criteria for disrupted representation in the alternative coding scheme. Using the disrupted classification may be particularly insightful in the current study given that previous work has suggested that caregiver unresolved trauma may be a significant correlate of disrupted classification (Crawford & Benoit, 2009).

Findings from the current study support that stressful life experiences are associated with how caregivers think and feel about their developing child. In particular, stressful life events may skew or distort caregivers’ views of their unborn child or distract them from considering the child and their relationship to them. Specifically, the role of stressful life events during pregnancy may be particularly relevant for the development of prenatal representations, evidenced by strong effect sizes. Categorical representations tend to be relatively stable pre- to postnatally (e.g. ranging from 62 to 80%; Benoit et al., 1997; Theran et al., 2005; Vreeswijk et al., 2015), and such postnatal representations have been linked with compromised caregiving, such as lower attachment security (Ahlfs-Dunn et al., 2022) and elevated negative mood during play interactions with infants (Korja et al., 2010). Thus, it is critical to understand precursors or correlates of prenatal representations in order to promote adaptive functioning for infants and the caregiver – child relationship. Future work exploring factors that may impact the stability of representations is critical for informing intervention and prevention efforts aimed at improving the quality of the caregiver – child relationship (e.g. parent gender, Branger et al., 2022). Findings from the current study highlight how stressful events, and in particular, stressful events during pregnancy, may be important for understanding beliefs and emotions that a pregnant person holds, with relevance for the caregiver – child relationship.

## Figures and Tables

**Table 1.** Summary of WMCI representation subscales.

Representation	Characterized by:
**Balanced Representations:**	
Balanced–Full	Exceptionally coherent, open, and rich. The child is cherished and clearly in-focus with little sense of distortion or anxious occupation.
Balanced–Restricted	Restriction in feeling about the child. The caregiver may be affectively muted or minimize the importance of the relationship with the child.
Balanced–Strained	Some strain is present in the caregiver’s relationship with the child. The caregiver may perceive the child as difficult or confusing, however, the caregiver is aware of this strain.
**Disengaged Representations:**	
Disengaged–Impoverished	A significant lack of the caregiver’s psychological involvement with the child. The caregiver seems largely indifferent to the child or may have an aversion to them.
Disengaged–Suppressed	Emotional constriction or aloofness pervades the interview. The caregiver has some thoughts and concerns about the child but maintains an unmistakable emotional distance.
**Distorted Representations:**	
Distorted–Distracted	An inability to focus on the child and their relationship with them. The caregiver may be focusing on a variety of concerns unrelated to the child.
Distorted–Confused	Marked incoherence in the form of the caregiver’s confusion and uncertainty about the child and their relationship with them.
Distorted–Role-Reversed	A desire in the caregiver for the child to bear an excessive psychological burden for the relationship. Usually desiring friendship with or compliance from the child.
Distorted–Self-Involved	An unmistakable sense of the caregiver’s preoccupation with self rather than with the child. The child is valued as a reflection of the caregiver, rather than as an individual.

Summaries from Zeanah et al. (1996) Working Model of the Child Interview Coding Manual.

**Table 2.** Descriptive statistics for continuous variables of interest.

Variable	Mean	SD	Range
Lifespan Stressful Events	5.93	3.67	0–18
Childhood Stressful Events	1.58	1.61	0–7
Pregnancy Stressful Events	0.26	0.55	0–3
Benevolent Childhood Experiences	9.27	1.37	0–10

**Table 3.** Bivariate Pearson correlations.

	1.	2.	3.	4.
1. Lifespan Stressful Events	–			
2. Childhood Stressful Events	.62**	–		
3. Pregnancy Stressful Events	.28**	.20**	–	
4. Benevolent Childhood Experiences	−.28**	−.20**	−.09	–

* *p* < .05.

** *p* < .01.Correlations for representations (i.e. 5–7) are not provided given they are mutually exclusive classifications.
